# Comparative assessment and monitoring of deterioration of articulatory organs using subjective and objective tools among patients with amyotrophic lateral sclerosis

**DOI:** 10.1186/s12883-019-1484-2

**Published:** 2019-10-19

**Authors:** Wioletta Pawlukowska, Bartłomiej Baumert, Monika Gołąb-Janowska, Agnieszka Meller, Karolina Machowska-Sempruch, Agnieszka Wełnicka, Edyta Paczkowska, Iwona Rotter, Bogusław Machaliński, Przemysław Nowacki

**Affiliations:** 1 Department of Medical Rehabilitation and Clinical Physiotherapy, Pomeranian Medical University, Żołnierska 54, Szczecin, 71-210 Poland; 2 Department of Neurology, Pomeranian Medical University, Unii Lubelskiej 1, Szczecin, 71-210 Poland; 3 Department of General Pathology, Pomeranian Medical University, Unii Lubelskie 1, Szczecin, 71-252 Poland

**Keywords:** Amyotrophic lateral sclerosis, Dysarthria, Speech disorders, Voice Handicap Index, Frenchay Dysarthria Assessment

## Abstract

**Background:**

Amyotrophic lateral sclerosis (ALS) is a fatal degenerative disease of a rapid course. In 25% of ALS sufferers, speech disorders occur as prodromal symptoms of the disease. Impaired communication affects physical health and has a negative impact on mental and emotional condition. In this study, we assessed which domains of speech are particularly affected in ALS. Subsequently, we estimated possible correlations between the ALS patients’ subjective perception of their speech quality and an objective assessment of the speech organs carried out by an expert.

**Methods:**

The study group consisted of 63 patients with sporadic ALS. The patients were examined for articulatory functions by means of Voice Handicap Index (VHI) and the Frenchay Dysarthria Assessment (FDA).

**Results:**

On the basis of the VHI scores, the entire cohort was divided into 2 groups: group I (40 subjects) with mild speech impairment, and group II (23 subjects) displaying moderate and profound speech deficits. In an early phase of ALS, changes were typically reported in the tongue, lips and soft palate. The FDA and VHI-based measurements revealed a high, positive correlation between the objective and subjective evaluation of articulation quality.

**Conclusions:**

Deterioration of the articulatory organs resulted in the reduction of social, physical and emotional functioning. The highly positive correlation between the VHI and FDA scales seems to indicate that the VHI questionnaire may be a reliable, self-contained tool for monitoring the course and progression of speech disorders in ALS.

**Trial registration:**

NCT02193893.

## Background

Amyotrophic lateral sclerosis (ALS) is a rapidly progressing and ultimately fatal neurological disease characterized by gradual degeneration of the upper and lower motor neurons. The etiology of ALS is multifactorial, affecting both neurons and glial cells [1]. Recent studies have shown a higher risk of developing ALS in individuals with the intermediate-length cytosine-adenine-guanine nucleotide repeat expansions in the ATXN2 gene [2]. Typical symptom of disease progression is bulbar dysarthria, which is the commonest form of speech impairment in ALS. Bulbar dysarthria is exhibited by 80% of the sufferers and contributes to a considerable decline in quality of life [3, 4]. In 25% of ALS patients speech deterioration is the first symptom of the disease [5].

Despite having little impact on physical health, impaired communication adversely affects mental and emotional wellbeing [6, 7]. Initial dysarthria is associated with shouting or singing disorders and minor speech problems [8]. The movements of the articulatory organs become slower. Weakness of facial muscles usually does not occur in the first phase of the disease [8]. Of all the articulatory organs the tongue is where the debilitating effects of ALS can be observed first. As the disease progresses it affects the perioral area and the jaw [9, 10]. Compared to healthy individuals, ALS sufferers while speaking demonstrate slower and prolonged jaw and lips movements [9–11]. It has also been established that the data obtained from the observation and analysis of the articulators efficiency can be utilized as a practical clinical marker for a reliable assessment of ALS progression and the extent of speech deterioration [12]. ALS individuals present a 30% reduction of the tongue mass and a decline in the mobility of the lower lip and jaw. These changes have a more severe adverse effect on speech intelligibility than a functionally debilitating defect of any other component of the articulatory system [12, 13]. Progression of dysarthria leads to impaired speech intelligibility and then finally to anarthria [7].

The speed of ALS progression is measured by means of various clinimetric scales. The most commonly used global assessment instruments comprise: the ALS Functional Rating Scale-Revised (ALSFRS–R), the Norris Scale, Appel the ALS Rating Scale or the ALS Severity Scale [14]. For the quantitative evaluation of the efficiency of the articulatory organs, Frenchay Dysarthria Assessment (FDA) is the most frequently recommended scale [6, 15]. Whereas, speech intelligibility can be measured using the Computerized Assessment of Intelligibility of Dysarthric Speech - Sentence Subtest [16]. The main objective of this software is monitoring the progression of speech impairment in patients staying at home. The system is endowed with a feature enabling to control speech quality during their telephone conversations with the therapist. This, however, is not recommended as a reliable clinical measure for the evaluation of speech deterioration in ALS [17]. An alternative tool used for the objective assessment of ALS progression is the Multi-Dimensional Voice Program, based on a multi-parameter acoustic analysis [18].

Relevant research and reliable tests allowing a quick assessment of speech quality by the patients themselves are still scarce. Existing studies indicate that such an assessment is possible but would require sophisticated software or instruments, which might be too costly for the patients or their families [6].

Voice Handicap Index (VHI) is an easy to perform and convenient tool for the assessment of speech impairment which can be applied by ALS patients themselves in their home environment [19]. Owing to this instrument people with ALS can subjectively evaluate the impact their deteriorating speech has on their subjective perception of a decline in quality of life.

The first goal of this study was to assess which domains of speech are particularly involved in ALS patients. The second aim of the investigation was to estimate possible correlations between subjective sense of speech disturbances experienced and self-assessed by ALS patients by the use of VHI scale and an objective analysis performed by an expert using the FDA.

## Methods

### Patients

The study was designed as a prospective, open-label, nonrandomized clinical trial in a single center for subjects with ALS. The trial (international number: NCT02193893) was approved by the Ethics Committee of the Pomeranian Medical University in Szczecin, and conducted in accordance with the Declaration of Helsinki in 2014–2018 [20]. Written informed consent was obtained from each participant. The ALS patients consecutively enrolled in the study had to meet the following inclusion criteria:

- age ≤ 65 years,
- probable or certain sporadic ALS form diagnosed in compliance with the El Escorial Revised Criteria,
- ability to express informed consent,
- mild to moderate disability documented by satisfactory bulbar and spinal motor functions (minimum score 3 on the ALSFRS–R scale for swallowing and 2 points for food preparation and walking- patients able to independently reach the research facility),
- forced vital capacity (FVC) result ≥50%.

The study enrolled 63 patients - 25 females and 38 males – aged between 27 and 65 years old (mean: 57.03 ± 15.75) with sporadic ALS [20] according to the El Escorial Revised Criteria [21]. The exclusion criteria involved individuals suffering from any concomitant conditions affecting speech quality as well as those aged over 65 years due to the age-related decline in speech [22].

#### Speech test – VHI questionnaire

International research projects have demonstrated that the VHI questionnaire is a reliable tool for the subjective evaluation of voice in ALS patients and that the scores it provides are consistent. The index is made up of 3 components, each dedicated to a different aspect: functional, physical and emotional. The functional subscale measures the extent of the influence of voice deterioration on the subject’s everyday functioning; the physical one allows the assessment of the articulatory organs efficiency, whereas the emotional component enables to monitor the subject’s emotional reaction to the progressive speech deterioration [23, 24]. The 30-item VHI questionnaire, which comprises 3 subscales of 10 items each: the physical (P) items, functional (F) items and emotional (E) items, is a recommended method of subjective assessment of the severity of speech disorders by ALS patients themselves. Cronbach’s alpha for the entire cohort was 0.95, indicating high internal consistency of the 30 items. The VHI is a valid and reliable instrument approved by the Agency of Healthcare Research and Quality in 2002 [25] and can be recommended for ALS individuals. The questionnaire is widely used throughout the world in a number of language versions including Persian, Croatian, Italian, Portuguese, Latvian, Greek, Polish and many others [26–31]. Based on a detailed analysis of the VHI scores the patients were divided into group I with slight speech quality disorders (0–30 score range) and group II with medium and deep disturbances in the quality of speech (31–120 score range). Such a division allowed us to compare the differences between mild, medium and deep disturbances in the quality of speech in adequately large groups.

#### Speech test – FDA

One of the most important objective tests for evaluating the articulatory organs is the Frenchay Dysarthria Assessment (FDA). The FDA is a standardized test which relies on a 9-point rating scale applied to a patient. It provides information based on the observation of oral structures, functions and speech. The test evaluates the following functions: reflexes, respiration, tongue, lips, the soft palate, larynx, intelligibility. A 5-point rating scale (a – e) is used for the assessment, where letter ‘a’ represents norm, ‘b’ mild severity, ‘c’ moderate severity, ‘d’ considerable severity, ‘e’ profound severity. FDA is also used to assess the severity of the articulatory organs disorders and to monitor the effects of the treatment [15]. The test was conducted by a clinical speech therapist with 12 years’ experience of treating neurological conditions, predominantly Parkinson’s disease (PD). The second edition of FDA utilizes the latest findings concerning motor speech disorders and their contribution to a neurological diagnosis. It has good feasibility (missing data < 5%), a high reliability of the total score (0.94), an excellent inter-rater agreement for the total score (0.96) and moderate to large construct validity for 81% of its items [15, 32].

The VHI and FDA tests were performed simultaneously to assess their possible correlation at the same stage of disease.

#### Statistical analysis

The first goal of the study was to assess which domains of speech are particularly involved in ALS patients. The second aim of the investigations was to estimate possible correlations between a subjective sense of speech disturbances experienced by ALS patients and an objective assessment performed by an expert. The null hypotheses were that (1) the respective speech domains are not significantly involved depending on the advancement of the disease and (2) there are no correlations between subjective and objective assessment of speech disturbances. The alternative hypotheses were that the speech domains involvement differs significantly depending on ALS stage and that there are significant correlations between subjective and objective assessment of speech impairment. The numerical variables were presented as mean (M) ± standard deviation (SD). Because of the non-normality of the distributions between variables, the data were compared between groups I, II using the nonparametric Mann-Whitney U test, while the nominative variables by Fisher’s exact test. Correlations between subjective and objective assessment of respective speech domains for whole examined population and within the groups were studied with Spearman rank correlation. *P* values smaller or equal to 0.05 were considered to indicate statistical significance. All statistical analyses were performed with STATISTICA 12.5 PL.

## Results

63 patients participated in the study and were evaluated using the FDA and VHI scale. After analyzing the results, the patients were divided into two groups depending on the obtained VHI score. Group I consisted of 40 patients with slight speech quality disorders (0–30 points), and group II consisted of 23 patients with mean and profound speech quality disorders (31–120 points). Table 1 presents the general characteristics of the studied population of ALS patients.

**Table 1:** General characteristics of the examined population

Parameters		All patients (*n* = 63)
Age (years; mean ± SD)		57.03 ± 15.75
Disease duration (years; mean ± SD)		2.10 ± 1.85
Sex	Females	25 (39.68%)
	Males	38 (60.31%)
Speech disorder according to VHI scale	Group I	40 (63.49%)
	Group II	23 (36.50%)

Table 2 illustrates the correlation between age, sex and duration of the disease within particular groups according to VHI scale. No significant differences between selected parameters were found.

**Table 2:** Characteristic of the groups

Characteristics	Group I (*n* = 40)	Group II (*n* = 23)	*p* value
Age (years, mean ± SD)	53.58 ± 8.1	53.04 ± 10.9	0.94^a^
Sex (female/male)	14/26	11/12	0.31^b^
Disease duration (years, mean ± SD)	2.19 ± 1.98	1.96 ± 1.60	0.75^a^

^a^ Mann-Whitney U test; ^b^ Fisher’s exact test

Abbreviations: *n* group size; *p* statistical significance; *SD* standard deviation

Table 3 illustrates the exacerbation of articulatory disorders in patients constituting groups I and II, measured by objective means of FDA. In group I subjects, the efficiency of the articulators was within normal limits (a), mildly (b) or moderately impaired (c) in 95 to 100% of patients, whereas in their counterparts from group II the impairment was of considerable (d) to profound degree (e) in 13 to 56,4% of patients.

**Table 3:** Performance of the articulatory organs in patients from groups I and II measured with the FDA test in relation to the severity of vocal impairment assessed by means of the VHI scale

VHI		Group I (*n* = 40)	Group II (*n* = 23)	*p* value^a^
FDA				
Cough	a	17 (42.5%)	2 (8,6%)	*p* = 0.00001
	b	14 (35%)	2 (8,6%)	
	c	8 (20%)	6 (26%)	
	d	1 (2.5)	9 (39,1%)	
	e	0 (0%)	4 (17,3%)	
Swallowing	a	22 (55.0%)	2 (8.6%)	*p* = 0.00009
	b	12 (30%)	5 (21.7%)	
	c	6 (15.0%)	10 (43.4%)	
	d	0 (0%)	5 (21.7%)	
	e	0 (0%)	1 (4.3%)	
Saliva control	a	20 (50.0%)	2 (8.6%)	*p* = 0.00012
	b	14 (35.0%)	5 (21.7%)	
	c	6 (15%)	10 (43.4%)	
	d	0 (0%)	5 (21.7%)	
	e	0 (0%)	1 (4.3%)	
Breathing	a	20 (50.0%)	4 (17.3%)	*p* = 0.00204
	b	15 (37.5%)	7 (30.4%)	
	c	5 (12.5%)	8 (34.7%)	
	d	0 (0%)	3 (13.0%)	
	e	0 (0%)	0 (0%)	
Lip movements	a	11 (27.5%)	1 (4.3%)	*p* = 0.00036
	b	20 (50.0%)	7 (30.4%)	
	c	9 (22.5%)	8 (34.7%)	
	d	0 (0%)	7 (30.4%)	
	e	0 (0%)	0 (0%)	
Soft palate	a	12 (30.0%)	0 (0%)	*p* = 0.00004
	b	20 (50.0%)	5 (21.7%)	
	c	7 (17.5%)	8 (34.7%)	
	d	1 (2.5%)	9 (39.1%)	
	e	0 (0%)	1 (4.3%)	
Length of phonation	a	11 (27.5%)	1 (4.3%)	*p* = 0.00171
	b	14 (35.0%)	6 (26.0%)	
	c	14 (35.0%)	8 (34.7%)	
	d	1 (2.5%)	8 (34.7%)	
	e	0 (0%)	0 (0%)	
Pitch	a	12 (30.0%)	0 (0%)	*p* = 0.00009
	b	21 (52.5%)	8 (34.7%)	
	c	6 (15.0%)	4 (17.3%)	
	d	1 (2.5%)	9 (39.1%)	
	e	0 (%)	2 (8.6%)	
Voice volume	a	12 (30.0%)	1 (4.3%)	*p* = 0.00317
	b	20 (50.0%)	8 (34.7%)	
	c	6 (15.0%)	7 (30.4%)	
	d	2 (5.0%)	5 (21.7%)	
	e	0 (0%)	2 (8.6%)	
Tongue movement	a	14 (35.0%)	0 (0%)	*p* < 0.00001
	b	19 (45.7%)	3 (13.0%)	
	c	7 (17.5%)	13 (56.5%)	
	d	0 (0%)	5 (21.7%)	
	e	0 (0%)	2 (8.6%)	
Words	a	38 (95%)	5 (21.7%)	*p* < 0.00001
	b	2 (5.0%)	9 (39.1%)	
	c	0 (0%)	4 (17.3%)	
	d	0 (0%)	4 (17.3%)	
	e	0 (0%)	1 (4.3%)	
Sentence	a	33 (82.5%)	2 (8.6%)	*p* < 0.00001
	b	5 (12.5%)	8 (34.7%)	
	c	2 (5%)	8 (34.7%)	
	d	0 (0%)	2 (8.6%)	
	e	0 (0%)	3 (13.0%)	
Spontaneous speech	a	27 (67.5%)	2 (8.6%)	*p* < 0.00001
	b	9 (22.5%)	1 (4.3%)	
	c	3 (7.5%)	9 (39.1%)	
	d	1 (2.5%)	7 (30.4%)	
	e	0 (0%)	4 (17.3%)	

^a^ Fisher’s exact test

Abbreviations: *n* group size; *p* statistical significance

Table 4 demonstrates the correlation between the objective and subjective assessments of articulatory disorders in respect to the entire cohort, established through the application of the physical, emotional and functional subscales of the VHI. The analysis of the relationship between evaluation of the articulators efficiency and the patients’ self-assessment of speech quality demonstrated positive moderate correlation in 5 domains and high correlation in another 8 ones. Regarding the relationship between the FDA and the physical subscale of the VHI a very strong positive correlation was found in 9 articulatory domains and in 4 others the correlation was high. The scores the emotional subscale of the VHI provided showed a very high positive correlation in 6 domains and high correlation in 7 domains, while in the case of the functional component scores the relevant numbers of domains were 7 and 6 respectively.

**Table 4:** General assessment of the correlation between performance of the articulatory organs measured by means of the FDA scale and the VHI based on the degree of speech disorder (comprising total 30-items questionnaire and 3 subscales: physical, functional and emotional) in ALS patients

VHI	total 120 items R Spearman	physical (P) R Spearman	functional (F) R Spearman	emotional (E) R Spearman
FDA				
Cough	0.7472^c^	0.7177^c^	0.7333^c^	0.6962^b^
Swallowing	0.7732^c^	0.7648^c^	0.7367^c^	0.7560^c^
Saliva control	0.7069^c^	0.7081^c^	0.6777^b^	0.7022^c^
Breathing (at rest)	0.5552^b^	0.5470^b^	0.5095^b^	0.5732^b^
Lips (at rest)	0.6404^b^	0.6126^b^	0.6010^b^	0.6688^b^
Soft palate (at rest)	0.7927^c^	0.7741^c^	0.7833^c^	0.7212^c^
Length of phonation	0.5992^b^	0.5984^b^	0.5217^b^	0.5861^b^
Pitch	0.6945^b^	0.7014^c^	0.5741^b^	0.6254^b^
Voice volume	0.6360^b^	0.6695^b^	0.6233^b^	0.5644^b^
Tongue (at rest)	0.7442^c^	0.7055^c^	0.7633^c^	0.7391^c^
Speech comprehension	0.7190^c^	0.7042^c^	0.7352^c^	0.6568^b^
Sentence comprehension	0.7871^c^	0.7882^c^	0.7670^c^	0.7275^c^
Spontaneous speech comprehension	0.7796^c^	0.7710^c^	0.7827^c^	0.7039^c^

Strength of correlation: ^a^weak positive; ^b^moderate positive; ^c^strong positive

Table 5 illustrates the intergroup correlation between the objective assessment of the efficiency of the articulatory organs and the subjective evaluation of speech performed by means of the FDA. Group I showed a high positive correlation in 5 articulatory domains and in 7 others the positive correlation was of an average intensity. As for group II, a very high positive correlation was observed in one domain, high in 8 and average in another 3 domains.

**Table 5:** Assessment of the correlation between performance of the articulatory organs measured by means of the FDA scale and the VHI based extent of vocal impairments in ALS patients divided into groups

VHI	Group I (*n* = 40) VHI total 120 items R Spearman	Group II (*n* = 23) VHI total 120 items R Spearman
FDA		
Cough	0.5499^b^	0.5200^b^
Swallowing	0.6762^b^	0.5542^b^
Saliva control	0.4871^a^	0.5983^b^
Breathing (at rest)	0.3881^a^	No correlation
Lips (at rest)	0.5003^b^	0.4997^a^
Soft palate at rest	0.6513^b^	0.5615^b^
Length of phonation	0.4956^a^	0.6106^b^
Pitch	0.4043^a^	0.7145^c^
Voice volume	0.4345^a^	0.6621^b^
Tongue (at rest)	0.4017^a^	0.5328^b^
Speech comprehension	No correlation	0.4333^a^
Sentence comprehension	0.4978^a^	0.5412^b^
Spontaneous speech comprehension	0.5370^b^	0.4928^a^

Strength of correlation: ^a^weak positive; ^b^moderate positive; ^c^strong positive.

Abbreviations: *n* group size

## Discussion

Speech disorders are one of the most common manifestations of ALS and a real challenge for both clinicians and researchers [33]. Early dysarthric symptoms occur sporadically and usually come in the form of hoarseness and declined speech at the end of the day. As the disease progresses, the symptoms become more severe - the muscles of the tongue, lips and larynx get weaker and slower, their movement amplitude decreases and speech intelligibility declines [34].

Some researchers argued that speech impairment in ALS is more prevalent in females [35]. Our study did not confirm the finding. We found that it was the male subjects who were affected by speech deficits more often (60.31%) than women. However, the difference was statistically insignificant and requires further in-depth studies to draw further conclusions.

Tongue dysfunction is commonly considered as one of the typical symptoms of speech deterioration in an early stage of ALS [36]. Other symptoms include a decline in the functionality of the soft palate, larynx and lips [36, 37].

Among the participants of our study who presented mild speech impairment established by means of the FDA scale, the most prevalent abnormalities were observed in tongue mobility (63.2%), soft palate (70%) and lips (72.5%). The obtained results correspond with the current state of knowledge on the subject [36, 37].

The main measure of communication efficiency in ALS subjects is the extent to which their speech is intelligible. Impaired speech undoubtedly affects quality of life and the severity of communication deficit is a determinant of the treatment option that ought to be applied [12]. Speech intelligibility could be treated as a practical measure used in monitoring the quality of articulation during the course of treatment as well as the assessment of the progression of the disease process [38].

Yunusova et al. demonstrated the existence of a direct relationship between speech intelligibility and the mobility of the lips and jaw in ALS subjects [36]. Other researchers reported lack of correlation between speech comprehension in ALS and progression of the disease [39]. It has also been found that in the early stages of the disease the reduced mobility of the tongue, lips and jaw does not affect speech intelligibility [40]. A considerable deficit in this respect, though, can be observed with the emergence of signs of critical laryngeal dysfunction [34].

Our study shows that the ALS patients presenting mild impairment of the articulatory system, measured using the objective FDA scale, do not perceive significant deterioration of speech intelligibility, established by means of the subjective VHI. It is not until the occurrence of a considerable decrease in the articulators efficiency that a substantial decline in speech becomes evident. We found that a speech intelligibility decline coincides with dysfunctions in the length of phonation, pitch and voice volume. Also, the study revealed the existence of a correlation between FDA-based assessment of speech deterioration and the patient’s perception of a decline in the physical and functional aspects of everyday life established by means of the VHI. These findings provide a valuable contribution to the knowledge of ALS progression and its impact on life quality of ALS sufferers.

Existing research does not mention any relationship between a progressive deterioration of motor functions and disorders of emotional nature [41]. Some earlier publications noted ‘the disability paradox’ phenomenon, a term that refers to the alleged lack of relationship between the subjective perception of physical dysfunction reported by ALS patients and a decline in quality of life [42, 43]. Others, on the other hand, pointed to a weak or moderate correlation between the disease severity and the emotional condition of the sufferer [44]. The latter pattern was reflected in our study – the participants complained of a depressed mood they experienced in line with the progression of speech deficits.

The progression of speech deterioration in ALS is very rapid, which is reflected by the fact that the median survival for the disease is merely 30 months [45]. It is therefore vital that the process of speech deterioration should be monitored in a clinical setting so that new communication strategies could be devised and introduced in time. However, the range and reliability of currently available instruments for adequate monitoring of the articulatory functions is still insufficient. The limitations mainly concern the number of factors affecting the accuracy of the relevant examinations, their validity and reproducibility. Normalized diagnostic procedures applied for the evaluation of articulation quality and monitoring speech regression in ALS are very scarce [39]. This can be attributed to the logistic difficulty connected with the analysis of bulbar symptoms. Shortages of proper tools for the assessment and monitoring of speech deterioration are commonplace in clinical environment. The procedures in most instances require the employment of sophisticated instruments such as tensometric sensors or air pressure bulbs, whose reliability and precision are yet to be properly documented [39, 46]. One of the earliest predictors of ALS progression is a decline in the movement velocity of the articulatory organs [39].

The tools that we applied in our study to monitor the efficiency of the articulators and speech intelligibility comprised the FDA and the VHI rating scales. The analysis of the relationship in respect to the entire cohort demonstrated positive moderate correlation in 5 domains and high correlation in another 8 ones. Regarding the relationship between the FDA and the physical subscale of the VHI a very strong positive correlation was found in 9 articulatory domains and high correlation in 4 others. The scores the emotional subscale of the VHI provided showed a very high positive correlation in 6 domains and high correlation in 7 domains, while in the case of the functional component scores the relevant numbers of domains were 7 and 6 respectively. In terms of correlation with the division into groups, group I showed a high positive correlation in 5 articulatory domains and in 7 others the positive correlation was of an average intensity. As for group II, a very high positive correlation was observed in one domain, high in 8 and average in another 3 domains. Summarizing, the extent of a decline in the performance of the articulatory organs established by an experienced researcher corresponded with the patient-reported self-assessment of speech comprehensibility. These findings were substantially different from the outcomes of another study we carried out on the subjective and objective assessment of the extent of articulatory disorders in PD patients [32]. It may be the case that Parkinsonian patients are well-adjusted to the changes in the articulatory system. It needs to be noted that the average duration of Parkinson’s disease is 15.8 years [47] compared to 30 months in the case of ALS [48]. Consequently, a dramatic speech deterioration in ALS is observed within a significantly shorter space of time. The results of this study imply that the VHI could be used as a reliable self-applied instrument for monitoring the course and progression of speech impairment in ALS as well as a prognostic instrument for predicting the progression of speech deterioration, which apart from being an early marker of acute speech exacerbation [39], is of critical importance in the context of therapeutic procedures for ALS. In the light of the abovementioned facts, it can be assumed that the VHI is a reliable marker of speech decline in ALS and thereby a useful tool for the verification of the onset of the symptoms and their progression, which will ultimately help to introduce proper therapeutic strategies. The application of a regular, cyclical examination of speech function using a self-assessment tool (VHI questionnaire) would have many advantages, i.e. the possibility of self-conducting a home examination, reducing the number of visits to a specialist with cost limitation, rapid screening of patients with deterioration of articulation requiring specialist assessment and treatment.

## Conclusions

The pathologic changes that occur in the tongue, lips and soft palate are typical features of the early symptoms of ALS. Individuals with ALS reported perceived speech decline at the time of the onset of phonatory impairment. A decline in the efficiency of the articulatory organs adversely affected social, physical and emotional functioning. A strong positive correlation was found between an objective evaluation of the articulators’ efficiency performed by a specialist using the FDA scale and a subjective self-assessment of the feature conducted by the patients utilizing the VHI scale. It can be concluded that the VHI constitutes a valid measure of the articulation disorders in ALS patients, contributing to the decision making process regarding the compensatory treatment options aimed at the articulators affected by the disease.

## Data Availability

The datasets used and/or analysed during the current study are available from the corresponding author on reasonable request.

## Abbreviations

ALS: Amyotrophic lateral sclerosis
ALSFRS–R: ALS Functional Rating Scale-Revised
FDA: Frenchay Dysarthria Assessment
FVC: Forced vital capacity
M: Mean
PD: Parkinson’s disease
SD: Standard deviation
VHI: Voice Handicap Index
